# Base of Skull Metastatic Adenocarcinoma from the Breast 23 Years after the Primary Diagnosis

**DOI:** 10.1155/2020/2610597

**Published:** 2020-07-30

**Authors:** Iylia Ajmal Othman, Farah Dayana Zahedi, Salina Husain

**Affiliations:** ^1^Department of Otorhinolaryngology Head and Neck Surgery, Kulliyyah of Medicine, IUniversity Malaysia, Kuantan, Pahang, Malaysia; ^2^Department of Otorhinolaryngology Head & Neck Surgery, Faculty of Medicine, Universiti Kebangsaan Malaysia, Kuala Lumpur, Malaysia

## Abstract

Breast cancer metastases to the base of the skull with concomitant infiltration into the paranasal sinuses and nasopharynx are exceptionally rare with only small numbers of reported literatures. Greenberg et al. in 1981 described five clinical syndromes with regards to the base of skull metastases and the clinical presentation of each syndrome related to its anatomical location. Often, metastases to the base of the skull remain asymptomatic until the lesion has increased to a considerable size causing bony destruction and impingement to the surrounding structures. When involving the paranasal sinuses or nasopharynx, the most common presenting symptoms mimic those of rhinosinusitis and, hence, may delay the accurate diagnosis. We are reporting a case of base of skull metastasis from breast carcinoma, 23 years after the primary diagnosis. To the best of our knowledge, our case is the first case to report latent metastasis of more than 20 years.

## 1. Introduction

Breast cancer metastasis to the head and neck region is uncommon with only few cases documented in the literature. Particularly, metastatic diseases to the base of the skull with infiltration into the nose and paranasal sinuses are exclusively rare [1–11].

Previous published papers reported that the mean duration from primary diagnosis of breast cancer to base of skull metastasis was 71 months [12] with an overall median survival of 31 months [13]. In 2013, Johnston et al. reported the longest duration of latent metastasis to the base of skull involving the paranasal sinuses from breast cancer primary, 20 years after the initial diagnosis [2].

In 1981, Greenberg et al. identified the five clinical syndromes with regards to skull base metastases and their incidences: the orbital (7%), parasellar (16%), middle-fossa (35%), jugular foramen (16%), and occipital condyle syndromes (21%) [1]. The clinical presentation of these syndromes is determined by the anatomic location of the lesion. When involving the orbits, the patient will present with diplopia, epiphora, blepharoptosis, decreased visual acuity, and proptosis [13]. Clinically, the most apparent presentation is progressive ipsilateral involvement of the cranial nerves [14]. Metastasis of the anterior skull base (involving the nose and paranasal sinuses) is very rare, and they may present as progressive periorbital swelling and epistaxis [14].

The prognosis of skull-base metastases depends on the nature and dissemination of the primary tumour, as well as the site, extension, and surgical resectability of the metastasis tumour itself. The development of skull-base metastases is often a late occurrence in the course of a cancer disease, and death is generally due to the systemic progression of the disease. Most patients with skull base metastasis were asymptomatic. However, some may present with local pain and involvement of cranial nerve palsies that harmed their quality of life. Treatment offered was mainly for symptomatic relief, by local or whole-skull irradiation [12].

We will be discussing the importance of heightened suspicion in a patient with a background history of carcinoma presented with multiple cranial nerve palsies and the treatment options, as well as the proposed mechanism contributing to the latent phase of disease presenting as late-onset metastasis, as seen in our case.

## 2. Case Report

In 2016, a 67-year-old lady with a known history of left breast cancer presented to us with a 3-month history of total loss of left eye vision, which was preceded by a 3-month history of progressive blurring of vision. A year prior to this presentation, she was diagnosed with left vocal cord palsy presented with hoarseness. With regards to her previous history of breast cancer, she underwent left mastectomy and axillary clearance and completed her radiotherapy back in 1992.

Clinical examination showed loss of left eye vision and absent corneal reflex, right eye lateral rectus palsy, hypoglossal nerve palsy, and left vocal cord palsy. Endoscopic examination showed a well-encapsulated mass at the superior aspect of the nasal cavity. Examination of the contralateral breasts revealed no palpable mass and no axillary lymphadenopathy. These findings correlated with her annual mammogram which showed no mammographic evidence of malignancy (BIRADS 1). Blood investigations prior to radiological investigation and in preparation for surgery were within normal limits.

**Figure 1:**
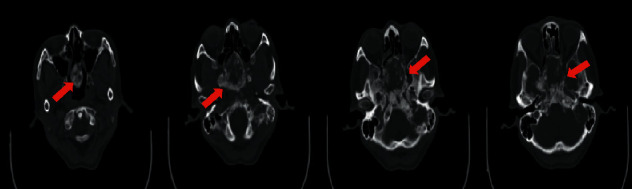
Showing bony destruction involving the anterior and middle cranial fossa floor, pituitary fossa, both lamina papyracea, medial part of the greater and lesser wing of sphenoid bone, wall of sphenoid sinus, and posterior nasal septum.

She underwent contrast-enhanced computed tomography (CECT) of the base of the skull, paranasal sinuses, until the neck region. The scan showed a large, lobulated, heterogeneously enhancing mass seen with epicentre at the sphenoid bone measuring 5.7 × 6.4 × 4.8 cm. Anteriorly, the mass occupied the posterior ethmoid sinuses. Laterally, there was bowing of the lamina papyracea bilaterally, indenting into extraconal spaces. Posteriorly, it extended to the prepontine cistern. There was bony destruction seen involving the anterior and middle cranial fossa floor, pituitary fossa, both lamina papyracea, medial part of the greater and lesser wing of sphenoid bone, wall of sphenoid sinus, and posterior nasal septum (Figure 1). Another similar heterogeneously enhancing lesion was seen within the left jugular fossa measuring 2.8 × 1.9 × 2.5 cm.

**Figure 2:**
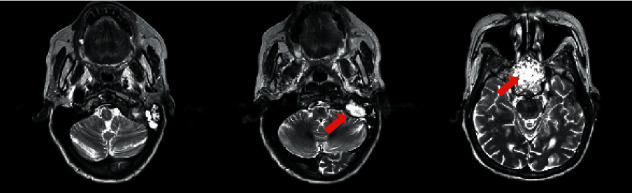
Anterior skull base tumour which has eroded into the sphenoid sinus occupying the entire sinus and another lesion seen within the left jugular fossa.

She also underwent Magnetic Resonance Imaging (MRI) with gadolinium-based contrast, which showed an anterior skull base tumour surrounding the olfactory nerve and has eroded into the sphenoid sinus occupying the entire sinus. A similar hyperintense lesion was seen within the left jugular foramen (Figure 2).

She was subsequently subjected for examination under anaesthesia and planned for biopsy. Intraoperative findings showed a lobulated, well encapsulated, mixed consistency (soft to firm) tumour occupying the posterosuperior part of the nasal cavity in the midline. Anteriorly, it involved the posterior part of the septum until the just anterior to sphenoid rostrum. Inferiorly, it bulged into the nasopharynx about 20%. The tumour appeared highly vascular and bled upon probing; hence, we were unable to proceed with tumour debulking.

Histopathological examination revealed metastatic adenocarcinoma suggestive of breast primary (estrogen-receptor positive, cytokeratin-7 positive, and thyroid transcription factor-negative). She, then, had a bone scan performed a month after the biopsy, which showed local bony destruction involving the clivus, bilateral petrous, bilateral anterior arc, and lateral mass of C1 vertebra. The treatment options offered were radiotherapy and chemotherapy. She completed 10 cycles of radiotherapy (total of 30Gy) and completed 6 cycles of chemotherapy. She was, afterwards, started on letrozole (aromatase inhibitor) and calcium supplement.

Approximately 20 months after the initial investigation, she underwent CT of the base of the skull which displayed no significant changes in the size and extension of the previously seen sphenoid mass. There was no mass in the left jugular fossa which was previously visualized. There was medial deviation of the left vocal cord possibly due to recurrent laryngeal nerve palsy secondary to vagus nerve compromise by either skull base metastasis or radiation therapy.

Unfortunately, she complained of dysphagia 10 months after completion of chemoradiotherapy. In view of failed FEES (fibreoptic endoscopic evaluation of swallowing), she was referred to the upper gastrointestinal team for percutaneous endoscopic gastrostomy (PEG) tube insertion.

During one of her outpatient appointments, 15 months after completion of chemoradiotherapy, she was generally well with no history of epistaxis and was on PEG tube feeding. She did not regain her left eye vision, solely using her right eye.

## 3. Discussion

More than half of the patients diagnosed with breast cancer will present with metastastic disease, but involvement of the head and neck is exceptionally rare. Only a small number of cases which metastasized to the larynx, nasopharynx, parotid gland, nose, and paranasal sinuses have been reported [15]. When involving the head and neck region, the common metastatic sites are the maxillary, ethmoid, frontal and sphenoid sinuses, and the nasal cavity, as well as the vascular channels [6, 16–19].

The most known primary tumors to potentially give metastases in this area are renal cancer which are usually highly vascularized and, hence, may present with epistaxis [4], followed by testicular tumors, bronchial cancer, gastrointestinal neoplasm, and breast cancer [15]. In the case of breast cancer primary, higher incidence is described in human epidermal growth factor receptor 2- (HER2-) positive as compared to HER2-negative patients [20].

As reported in the previous literatures, metastatic cases to the skull base and paranasal sinuses from primary breast cancer had very poor prognosis, and treatment plans were generally palliative to alleviate symptoms. Radiotherapy is the standard treatment usually offered with some patients also benefitting from chemotherapy [11, 12]. Xiong et al. reported a triple-negative breast cancer with metastasis to the paranasal sinuses. Despite being associated with the poorest prognosis, their case showed no progression of disease during the 32 months of observation. They attributed this to the effectiveness of combined modality of treatment with chemotherapy and radiotherapy. They suggested the docetaxel combined with cisplatin regimen as the choice of chemotherapy [11]. This combined modality of treatment was similarly prescribed in our case with subsequent continuation with oral letrozole.

Surgical resection or debulking is also offered to some patients with the aim to provide symptomatic relief and preserve function. It can also be the method to gain tissue biopsy for histological assessment. Palliative surgical debulking may be the treatment option in radiotherapy-resistant tumours which present with worsening neurological deficits [21]. Unfortunately, in our case, the nature of the tumour which was highly vascularized hindered this treatment option. Nevertheless, we managed to get sufficient tissue samples for histological assessment, thus arriving to the diagnosis of metastatic adenocarcinoma from breast cancer primary.

In a study by Mitsuya et al. in 2011, they found breast cancer as the most common primary site (55%) with the mean time from primary diagnosis to skull metastasis diagnosis of 71 months [12]. A review by Laigle-Donadey and colleagues found an overall median survival of those diagnosed with metastasis skull-base tumour of 31 months [13]. Survival may be much longer in patients with breast cancer, justifying vigorous treatments that can be offered in selected patients. In their review, breast cancer was associated with the best survival of 60 months. Conversely, the presentation of cranial nerve palsies indicates a poorer prognosis with an average survival of only 5 months after the onset of cranial nerve involvement.

Patients with metastatic lesions in the area of the base of the skull with paranasal sinuses involvement have no distinctive features and maybe discovered incidentally on routine imaging for cancer staging or during brain imaging for head trauma or other head and neck pathology. The affected patients may remain asymptomatic for a long period; hence, early diagnosis is considered difficult. In the literature, the interval between the diagnosis of breast cancer and paranasal metastasis has been reported to range from 3 months to 20 years [2, 3]. There was no reported case of base of skull metastasis from the breast for more than 20 years. To the best of our knowledge, our case is the first case to be reported.

They may initially present with subtle manifestation mimicking that of acute or chronic rhinosinusitis, nasal blockage, and recurrent epistaxis. Some may mimic primary orbital or maxillofacial malignancy. When involving the orbits, the patient will present with diplopia, epiphora, blepharoptosis, decreased visual acuity, and proptosis [4, 22]. Although the diagnosis of base of skull metastasis is usually made in patients with known primary, in approximately 28% of cases, these lesions can be the first presentation of cancer [13].

The attending surgeon or physician must be extravigilant when faced with patients with a known primary cancer who presented with cranial neuropathy or craniofacial pain. Base of skull metastasis should be strongly suspected. The clinical presentation is typically determined by the anatomic location of the lesion and may only become clinically apparent when the lesion has increased to a considerable size and directly affecting the adjacent structures [13].

Distant metastatic spread is unusual before the disease spreads locally, and distant metastases are uncommon in the absence of lymph node metastases. In most cases of head and neck metastases, the route of cancer dissemination is thought to be hematogenous [22]. Haematogenous spread to the skull or the meninges is more frequently seen in lung cancer primary [5].

The postulated mechanism of cancer dissemination is via retrograde venous seeding along the extensively interconnected midline Batson's valveless venous system from pelvic structures to the basilar plexus of veins, seen mainly in the case of prostatic cancer [21].

Specifically, in the case of breast cancer primary, the route of spread from the infiltrating ductal carcinoma of the breast to the sphenoid sinus is suspected to be hematogenous, while symmetrical ethmoid metastasis is suggested to be via transcribrosal spread [3, 14].

Batson, in 1940, performed an experiment to assess the venous spread of disease from breast cancer. He injected mercury sulphide into a small vein in a cadaver's left breast. The injectable material was, then, found in the clavicles, in the intercostal veins, the head of the humerus, the cervical vertebrae, and the transverse cranial venous sinus, as well as in the superior longitudinal sinus. Some of the material were also found in the azygous vein and in the superior caval system. These replicated observations corresponded to the so-called aberrant metastases from the breast, to the paranasal sinuses, the skull bones, the cervical vertebrae, and to the shoulder girdle [23].

## 4. Conclusions

Metastasis to the base of the skull with concomitant paranasal sinuses involvement, as seen in our case, is exclusively rare. Despite the rarity of this disease progression, the attending physician and surgeon should have heightened suspicion of possible base of skull metastasis, especially when patients with a known primary cancer present with intractable epistaxis or symptoms mimicking rhinosinusitis, which are not responding to standard treatment.

The discernable feature of our case is the more than two decades of asymptomatic interval from the primary diagnosis of breast cancer. Therefore, latent metastasis to the base of the skull with concomitant infiltration to the paranasal sinuses or nasopharynx should not be ruled out although the primary cancer was diagnosed two decades ago. To date, our patient remains reasonably well more than 40 months after the diagnosis of skull base metastasis despite multiple cranial nerves involvement, although she requires PEG tube feeding and permanently lost left eye vision.
